# Why we postpone sleep: bedtime procrastination beyond self-control failure

**DOI:** 10.1093/sleep/zsag061

**Published:** 2026-03-06

**Authors:** Sooyeon Suh

**Affiliations:** Department of Psychology, Sungshin Women’s University, Seoul, Republic of Korea; Human-centered AI Institute, Sungshin Women’s University, Seoul, Republic of Korea

Since its introduction into the sleep literature [1], bedtime procrastination—defined as the voluntary delay of bedtime in the absence of external constraints—has become an increasingly visible feature of modern sleep behavior and a commonly cited explanation for insufficient sleep. This concept has become especially pertinent given accumulating evidence linking bedtime procrastination to poor sleep health [2–6]. However, despite mounting evidence, bedtime procrastination has remained a marginal concept within traditional theories of sleep timing and regulation, leaving many questions unaddressed. Is this behavior merely a failure of self-regulation, or a passive response to modern environmental cues that influence our circadian rhythm? Can it even reflect an adaptive attempt to delay bedtime awaiting a more optimal sleep window, when pre-sleep arousal decreases? Furthermore, it remains unclear how these delays in bedtime integrate with established circadian sleep science—specifically, whether they represent a stable phase-relationship or a state of chronic circadian misalignment.

In this issue of *SLEEP*, Kok et al. [7] advances the concept of bedtime procrastination by examining bedtime procrastination on a more granular, day-specific level, using daily diaries paired with actigraphy to capture its effects on next-day mood and sleepiness in a sample of residential college students. Consistent with previous studies [2], bedtime procrastination was a frequent behavior reported by the collegiate population, with students going to bed later than intended on 54% of all nights, and 99% of students engaged in bedtime procrastination at least once in 2 weeks. Furthermore, delaying bedtime was associated with a twofold increase in the odds of next-day daytime sleepiness and significant increases in negative affect alongside decreases in positive affect, suggesting that bedtime procrastination may contribute to persistent sleep insufficiency with implications for mental health in college students. Taken together, bedtime procrastination is not only a high-frequency behavior but also one that is associated with substantial risks to sleep and mental health.

One notable finding in the study challenges the common assumption that college students can readily catch up on sleep because of their supposedly flexible schedules. Objective actigraphy data revealed that students lost up to 50-60 min of sleep on both school and non-school nights because wake-up times remained relatively fixed, indicating that students do not compensate for bedtime procrastination by waking up later. While these fixed wake-up times may have been caused by external events, it is also possible this behavior aligns with a behavior-centric model of entrainment previously proposed by the authors elsewhere [8]. Based on this model, internal timing is shaped by social cues (such as class schedules) and self-selected light–dark cycles rather than solely on solar time. Thus, within this framework, bedtime procrastination is not merely a passive delay, but a behavioral decision that may actively reshape biological light–dark cycles.

Furthermore, results from this study indicated that daily bedtime procrastination predicted sleep loss, regardless of chronotype. Although evening types are generally more prone to late-night activities and may be more likely to engage in bedtime procrastination, the observed association was evident across chronotypes, regardless of their Morningness-Eveningness Questionnaire scores. These results move away from trait-level explanations to momentary processes, and reframes bedtime procrastination as a dynamic behavior rather than a stable individual difference. Most individuals who engage in bedtime procrastination deliberately postpone their bedtime despite knowing there will be next-day consequences to face; however, the behavior persists because these negative consequences are temporally discounted at the moment of decision, as short-term or unmet psychological needs are prioritized over delayed goals for getting sufficient sleep and preserving sufficient sleep and productivity.

Understanding bedtime procrastination as a momentary and dynamic behavior enables a more nuanced account into the diverse motivations of why an individual might engage in bedtime procrastination beyond framing it solely as a self-regulation failure. Taking a functional approach to bedtime procrastination to identify unmet psychological needs that drive the behavior can also guide personalized interventions to improve sleep in clinical settings. A functional approach addresses problematic behaviors by identifying the underlying contingencies that reinforce and maintain them. A qualitative study by Nauts and colleagues [9] categorized diverse reasons for bedtime procrastination into deliberate procrastination, mindless procrastination, and strategic delay. Deliberate procrastination is also often referred to as “revenge bedtime procrastination,” which involves willfully postponing sleep to reclaim personal freedom at the end of their day. This may differ from mindless procrastination, in which individuals lose track of time while immersed in an evening activity. This is often the case with college students, as a recent study found that they often do not plan their bedtime and frequently overrun their planned time, even if they do [10]. A strategic delay in bedtime may be particularly relevant to clinical populations such as insomnia patients, who often experience high pre-sleep arousal and regard bedtime as aversive due to unwanted nocturnal wakefulness and frustrated attempts to sleep. Our research also identifies emotion regulation, compensating for a long day (rewarding oneself), and the need for social connection as the top functions of bedtime procrastination in a sample primarily composed of college students [11]. Similarly, in the study by Kok et al. [6], losing track of time (32.6%) was the most common reason, followed by wanting “me” time (20%). While fewer studies have been conducted with clinical populations, our work with insomnia patients [12] suggests that while they share motivations of emotion regulation and compensation, “sleep induction”—delaying sleep until sufficient sleep pressure is accumulated—also emerged as a unique top reason. A move away from conceptualizing bedtime procrastination as a binary “self-regulation failure” enables a multidimensional perspective that reveals how individualized and momentary motivations can actively shift and reshape the circadian clock, thereby influencing next-day health. Next steps for future studies should further explore which subgroups are most vulnerable and when specific environmental and/or social antecedents are more likely to lead to bedtime procrastination to guide personalized sleep interventions.

Few sleep-related behaviors are as paradoxical as bedtime procrastination, which captures the intention-behavior gap: individuals report valuing sleep yet repeatedly delay it. As the work of Kok et al. demonstrates, bedtime procrastination is not merely a trendy construct or lifestyle choice that reflects self-regulation failure, but a modifiable bedtime behavior that causes significant sleep loss and next-day impairment. Considering that most current state-of-the-science sleep interventions were developed before modern devices that actively interfere with our sleep were invented, perhaps it may no longer be sufficient to encourage individuals to simply sleep earlier or more. Further examination into the pre-sleep period will be essential in ensuring that our behavioral choices also align with our biological rhythms.
